# Long-lasting negativity in the left motoric brain structures during word memory inhibition in the Think/No-Think paradigm

**DOI:** 10.1038/s41598-024-60378-y

**Published:** 2024-05-13

**Authors:** Viktoriya Vitkova, Dominique Ristori, Guy Cheron, Ariane Bazan, Ana Maria Cebolla

**Affiliations:** 1 Laboratory of Neurophysiology and Movement Biomechanics, Université Libre de Bruxelles, Brussels, Belgium; 2 InterPsy Laboratory, Université de Lorraine, Nancy, France

**Keywords:** Think/No-Think, Memory inhibition, Event related potentials, swLORETA, Neuroscience, Psychology

## Abstract

In this study, we investigated the electrical brain responses in a high-density EEG array (64 electrodes) elicited specifically by the word memory cue in the Think/No-Think paradigm in 46 participants. In a first step, we corroborated previous findings demonstrating sustained and reduced brain electrical frontal and parietal late potentials elicited by memory cues following the No-Think (NT) instructions as compared to the Think (T) instructions. The topographical analysis revealed that such reduction was significant 1000 ms after memory cue onset and that it was long-lasting for 1000 ms. In a second step, we estimated the underlying brain generators with a distributed method (swLORETA) which does not preconceive any localization in the gray matter. This method revealed that the cognitive process related to the inhibition of memory retrieval involved classical motoric cerebral structures with the left primary motor cortex (M1, BA4), thalamus, and premotor cortex (BA6). Also, the right frontal-polar cortex was involved in the T condition which we interpreted as an indication of its role in the maintaining of a cognitive set during remembering, by the selection of one cognitive mode of processing, Think, over the other, No-Think, across extended periods of time, as it might be necessary for the successful execution of the Think/No-Think task.

## Introduction

There is a wealth of evidence indicating common neural mechanisms underlying both the control of motor actions and the control of mental actions. For example, it is largely recognized that the mental simulation of a given action during motor imagery^1,2^ engages common brain networks to those underlying the real movement production^3–10^.

Analogously, there is consistent evidence that stopping motor actions and stopping thoughts may involve similar inhibitory cortical and subcortical mechanisms^11–13^. Stopping thoughts, and concretely stopping memory retrieval, can be assessed through the Think/No-Think (TNT) paradigm initially developed by Anderson and Green^14^. This paradigm consists of three phases: (1) the learning (memorization and recall tasks), (2) the TNT and (3) the final recall. During the learning phase, participants memorize a list of cue–target word pairs. During the TNT phase, stimuli are randomly attributed to either Think (T), No-Think (NT) or Baseline (B) conditions. In each trial, the cue word is presented, and participants must either remember the target word (T condition) or actively suppress any thought about the target by inhibiting memory retrieval (NT condition). It is generally admitted that if an item has already been committed to long-term memory, then its representation cannot be readily erased, and the NT instruction causes the suppression or inhibition of such representation^12^. During the final recall phase, all cue words are presented, and participants must recall the corresponding targets. The number of correctly recalled word pairs is measured. The standard finding is that recall is worse for items that were presented in the NT (this is called the suppression induced forgetting (SIF) effect), compared to the Baseline condition, the latter constituting a control condition for excluding simple forgetting over time. Additionally, T items are better recalled, compared to B items (T > B > NT).

Neuroimaging studies with functional magnetic resonance imaging (fMRI) have repeatedly reported right frontal cortex involvement during the TNT paradigm (right middle frontal gyrus^13^, right dorsolateral prefrontal cortex (DLPFC)^15^, ventro-prefrontal cortex (VLPFC), anterior cingulate cortex, pre-SMA, right parietal region^11^). The involvement of the right DLPFC and VLPFC in preventing a thought from coming to mind elicited by the TNT paradigm has been proposed as the same stopping mechanism marker as the one in stopping actions elicited by the stop signal paradigm^11,16^. This view supports the idea of direct suppression-induced forgetting produced by a top-down inhibitory control of the hippocampal role for thought retrieval^17–19^. Under this perspective, increased lateral prefrontal cortex activity correlates with decreased medial-temporal lobe and hippocampal activity in NT trials indicating that LPFC–hippocampal/MTL interactions lead to successful suppression^13,19–21^. It is important to note that in these studies, TNT instructions and memory cues (words) were simultaneously delivered. This implies that it was not possible to disentangle the effects related to the TNT instructions and to the memory content of the word cue.

Different from fMRI analysis, the features of event-related potentials (ERP) reflect direct measures of neural correlates, with excellent temporal resolution, of the (global) real electrical brain responses. The TNT paradigm has allowed to show that electrical slow brain responses of ERPs, localized over frontal and parietal scalp regions, are sensitive markers for the inhibitory control mechanisms of memory retrieval and that variations of such slow or late ERP component may underlie subsequent forgetting^18,22–24^. To the best of our knowledge, no ERPs studies have addressed the localization of the brain generators of such slow, late ERP component elicited by the word memory cues presentation.

The present study investigates the ERP’s electrical brain response elicited by the word memory-cues in the TNT paradigm. Conceptually following Hanslmayr et al.^22^, the presentation of the T/NT instructions and the word memory cues was dissociated by presenting the T/NT instructions continuously on the screen (for 1.5 s in the current study, for 1 s in Hanslmayr et al.^22^) before the onset of the word memory cues. This temporal segregation has previously allowed to differentiate an anticipatory executive control via top-down driven suppression mechanisms from the actual down-regulation of storage-related brain systems; this latter reflecting stronger directed suppression of the memory item itself, following the memory-cue^22,25^. The neural correlates of such temporal segregation corresponded to a first frontoparietal ERP positivity following the TNT instruction and a stronger frontoparietal ERP positivity following the memory cue. Our study focused specifically on the effects of the presentation of the word memory cue. Firstly, based on previous studies^22–24^, we expected to highlight sustained and reduced brain electrical frontal and parietal late potentials elicited by memory cues following the NT instructions as compared to T instructions. In a second step, we investigated the brain generators of such slow, late ERP components related to memory cues in the T and NT conditions by using standardized weighted Low-Resolution Brain Electromagnetic Tomography (swLORETA^26^). swLORETA is a distributed linear solution that does not initially preconceive the number or the location of the calculated generators in the gray matter volume^27–29^. To the best of our knowledge, no ERP studies have addressed the localization of the brain generators of such slow, late ERP component elicited by the presentation of the word memory cues.

## Results

### Behavioral

Friedman’s test was performed to examine how the three experimental conditions (T, NT, B) affect the number of correctly recalled words during the final recall task. The analysis did not show statistically significant differences between the three experimental conditions, χ^2^(2) = 0.62, *p* = 0.73. Pairwise comparisons were conducted using the Durbin-Conover test (NT vs. B: Z = 0.28, *p* = 0.78; T vs. B: Z = 0.49, *p* = 0.62; T vs. NT: Z = 0.77, *p* = 0.44). The recall accuracy at final recall (Mean% ± SD) was 95.40 ± 7.12 for B, 95.60 ± 6.95 for NT and 96.80 ± 5.60 for T. Additional descriptive statistics are available in Supplementary Tables 1–5.

### ERP analysis

**Figure 1:**
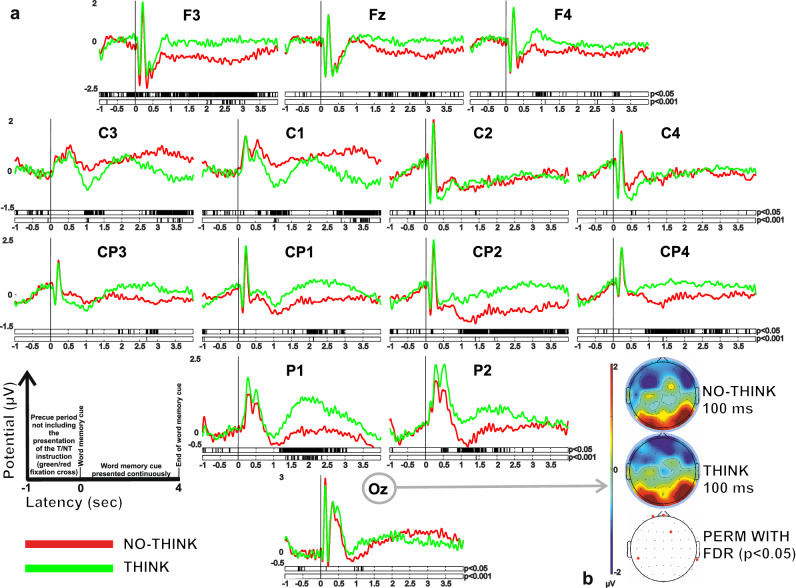
(**A**) Grand average ERP waveforms recorded at frontal, central, central-parietal, parietal and occipital scalp sites as a function of experimental condition (Think and No-Think in green and red curves respectively). The period from − 1 to 0 s corresponds to a truncated segment of the precue period that does not include the presentation of the green/red fixation cross announcing the T/NT instruction respectively, the period from 0 to 4 s corresponds to the entire duration of presentation of the word memory cue. (**B**) Topographical map showing electrical activity over the scalp at 100 ms after memory cue presentation.

Figure 1A shows the ERP grand averages in a scalp array of a set of illustrative electrodes for the T (green line) and NT (red line) conditions throughout the entire duration of the trial (− 1000–4000 ms). The period from − 1000 ms to 0 ms corresponds to a truncated segment of the precue period, not including the presentation of the green/red fixation cross announcing the T/NT instruction respectively, the period from 0 to 4000 ms corresponds to the entire duration of presentation of the word memory cue. Statistical differences in the population between the conditions and along time are represented by black bars in the lower part of each graphic (*p* < 0.05 and *p* < 0.001 in the lower line).

While T condition presented sustained electrical potential positivity over frontal, central-parietal and parietal scalp areas elicited by the memory cues, the NT condition presented maintained reduced brain electrical frontal and parietal potentials (F3: 0.17 ± 0.82 µV, 736 ± 101 ms; C1: 0.78 ± 1.75 µV, 507 ± 142 ms; CP2: − 0.34 ± 1.80 µV, 378 ± 108 ms; P1: 0.03 ± 1.80 µv, 880 ± 62 ms, in the T condition; F3 − 0.35 ± 0.70 µV, 740 ± 99 ms; C1: 1.01 ± 1.52 µV, 521 ± 152 ms; CP2: − 0.38 ± 2.58 µV, 332 ± 121 ms; P1: − 0.47 ± 1.41 µV, 914 ± 81 ms in the NT condition). Such stronger negativity of electrical potential took place relatively late after memory cue presentation at around 800 –1000 ms and lasted throughout the duration of the trial in frontal left areas (F3, where its significance is still present at *p* < *0.001*) and central parietal areas (CP2). Note that the significant differences appeared very early at the onset memory cue in F3 electrode. Both conditions presented similar electrical potentials in the occipital site (Oz: 2.90 ± 1.90 µV, 130 ± 13 ms in the T versus 3.25 ± 2.35 µV, 134 ± 11 ms in the NT condition; related topographical maps are illustrated in Fig. 1B).

**Figure 2:**
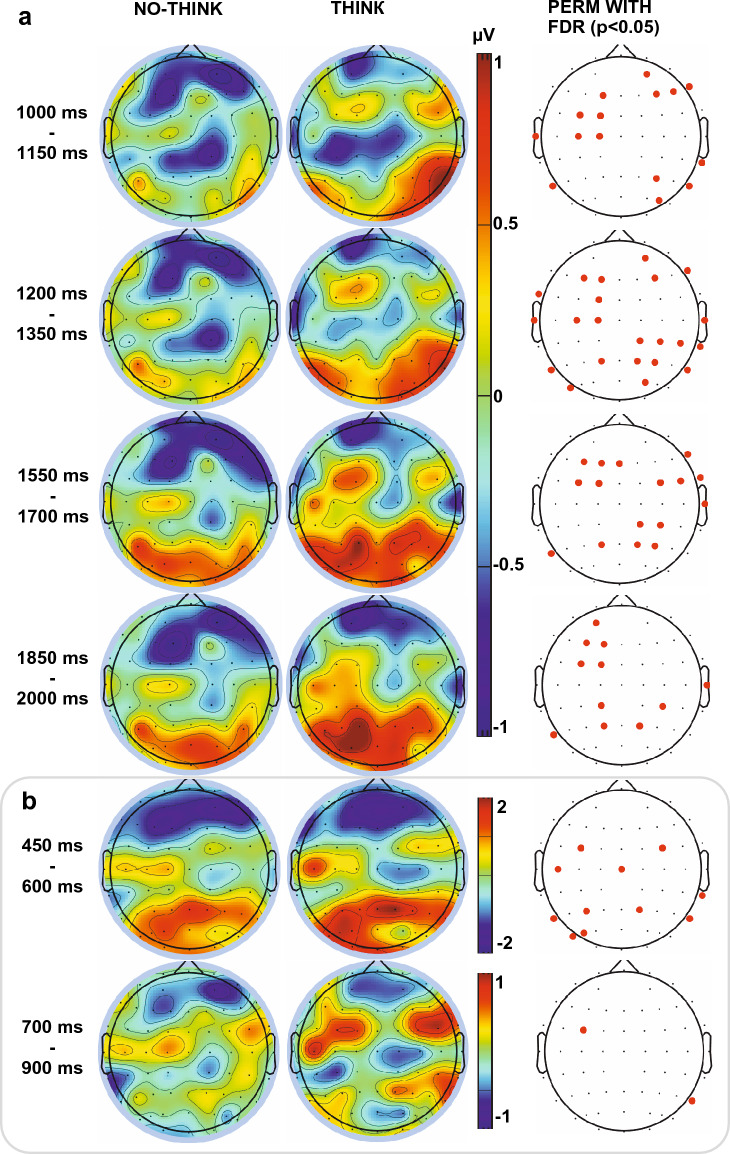
(**A**) Grand average topographical maps of electrical potential over the scalp in the No-Think (left) and Think (middle) conditions with their population statistical maps of differences (right) at time intervals 1000–1150 ms, 1200–1350 ms, 1550–1700 ms, 1850–2000 ms after memory cue presentation. (**B**) Grand average topographical maps showing electrical activity over the scalp in the No-Think (left) and Think (middle) conditions with their population statistical maps of differences (right) at time intervals previously reported as periods of interest^18^.

Figure 2A illustrates the grand average topographical distribution maps of electrical activity over the scalp for the NT (left column), T (middle column) conditions and their population statistical maps of significant differences (right column) at the following relevant periods : 1000–1150 ms, 1200–1350 ms, 1550–1700 ms, 1850–2000 ms. Topographical statistics did not reveal consistent spatially gathered populations of electrodes before 1000 ms nor after 2000 ms. It can be observed that the reduction of positivity exhibits two separate clusters at the frontal and central-parietal scalp areas in the NT condition with respect to the T condition. The grand average topographies in the T condition present several separate clusters of positive electrical potential bilaterally at frontocentral areas and bilaterally at parieto-occipital areas of the scalp. The statistical maps show differences situated in left frontal and central-frontal clusters, as well as at right parietal locations. Additionally, Fig. 2B illustrates the grand average topographical maps showing electrical activity over the scalp in the NT and T conditions with their population statistical maps of differences at time intervals 450–600 ms and 700–900 ms, previously reported as periods of interest^18^. Note that the frontal negativities observed in both conditions were not significantly different in the 450–600 ms period and that no consistent spatially gathered population of electrodes was revealed by the topographical statistics during the 700–900 ms period.

### Sources modelling

**Figure 3:**
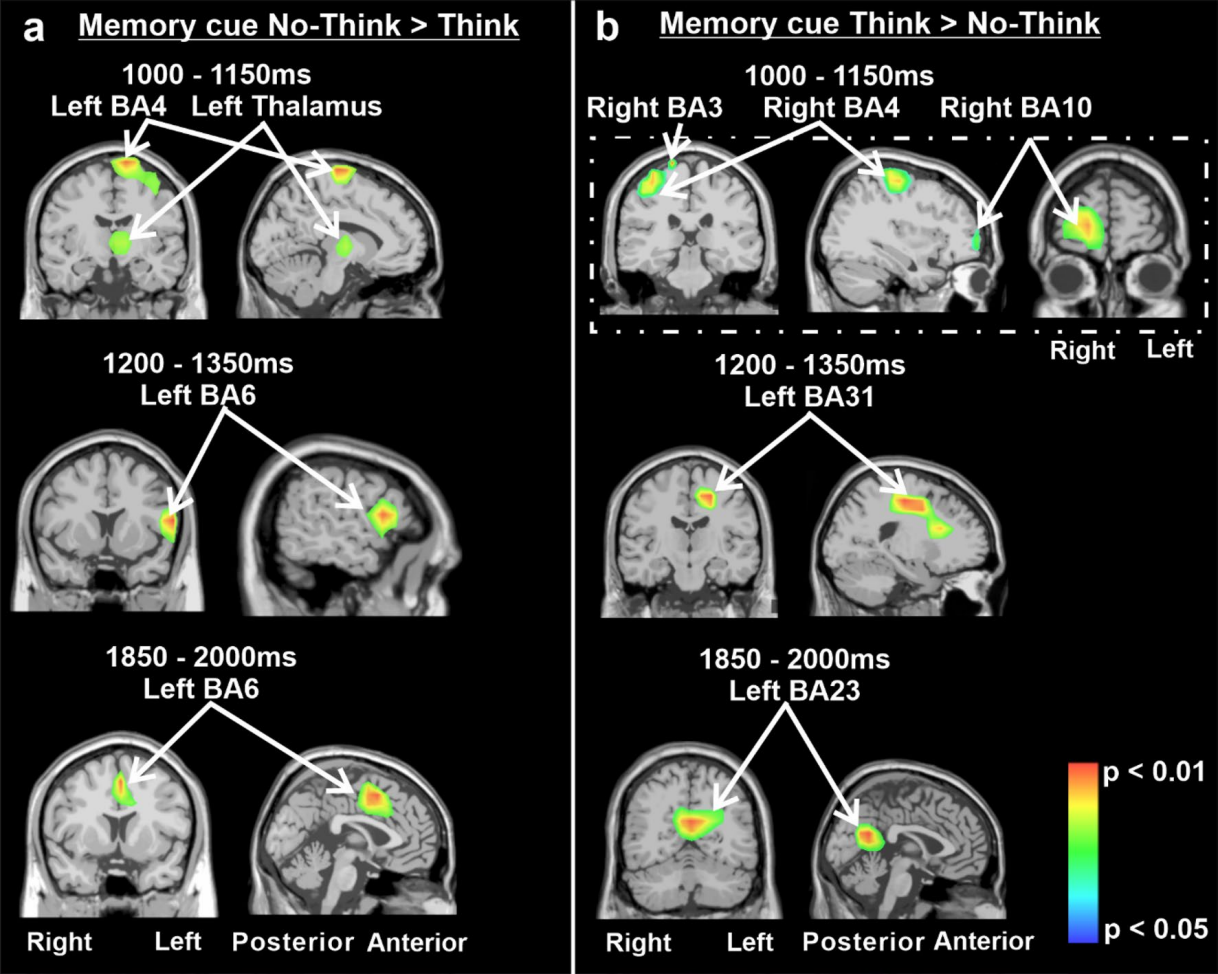
(**A**) Non-parametric statistical maps of the ERP sources for the No-Think > Think contrast at 1000–1150 ms, 1200–1350 ms, 1850–2000 ms after memory cue presentation. (**B**) Non-parametric statistical maps of the ERP sources for the Think > No-Think contrast at 1000–1150 ms, 1200–1350 ms, 1850–2000 ms after memory cue presentation.

Figure 3A and B illustrate the nonparametric statistic maps representing the estimated specific brain sources for the **No-Think** > **Think** contrast (A) and for the **Think** > **No-Think** contrast (B) for the periods of interest 1000–1150 ms, 1200–1350 ms, 1850–2000 ms after memory cue presentation (respectively in the upper, middle and lower rows). For the **No-Think** > **Think** contrast the model revealed two clusters situated in the left motor cortex (BA4, − 15, − 24, 71) and left thalamus (− 4, − 12, 3) during the first temporal period. In addition, the left premotor cortex was mainly involved for the next 1200–1350 ms and 1850–2000 ms periods with respective generators situated in BA6 (at − 58, 4, 14 and at − 2, 2, 48, respectively). For the **Think** > **No-Think** contrast the model revealed right sensorimotor involvement with clusters situated at the somatosensory cortex (BA3, 18, − 32, 70) and motor cortex (BA4, 37, − 22, 55) during the 1000–1150 ms period. Interestingly, during the same period, the right dorsolateral prefrontal cortex was involved with a cluster situated at BA10 (15, 55, 9). Later, during the 1200–1350 ms period, the model revealed the involvement of the left cingulate gyrus (− 17, − 23, 46) in BA23 and posterior cingulate cortex in BA31 (0, 59, 13) during the 1850–2000 ms period.

## Discussion

The aim of the current study was to investigate the topographical brain electrical potentials (ERP) elicited by the presentation of a word memory cue, following the previous instruction to either think about (remember) or inhibit (forget) a target word. Based on the original work of Hanlsmayr et al. ^22^, the present study adds the investigation of the estimated brain generators of the topographical ERP significant differences at long latencies (later than 1000 ms) elicited specifically by a word-memory cue after the TNT instructions by using a distributed source model which does not preconceive any spatial information within the gray matter volume. To the best of our knowledge, the present study involves the highest number of participants (forty-six) ever considered in an investigation of the neural correlates measured with high-density (64) EEG signals, of the thinking and not thinking of a word target provided by a memory word cue.

Interestingly, although the present results did not show significant behavioral differences between the thinking and not thinking of the target word, they revealed significant differences in their respective neural correlates which is in agreement with previous ERP studies^30^, demonstrating sustained and reduced brain electrical frontal and parietal late potentials elicited by memory cues following the NT instructions as compared to T instructions^18,22–24,31,32^. This pattern has been proposed as a marker of the reduced recollection activity in the NT^13,33^. Our topographical analysis revealed that such reduction was significant 1000 ms after memory cue onset and that it was long-lasting for 1000 ms. Our results are in line with the positivity reduction at around 1.6 s after memory cue presentation, which has been related to item suppression and thus to underlie the subsequent forgetting^22^.

In continuation of their previous ERP study^22^, Waldhauser et al.^25^ investigated the brain oscillatory dynamics and the related brain generators. They showed that compared to the T cue, the NT cue presented higher prefrontal theta power accompanied by fronto-parietal alpha phase synchronization. The successful suppression of target memories following the reminder presentation was characterized by a sustained and widespread decrease of theta oscillatory power spectrum situated in the medial temporal lobes and a reduced long-range theta phase synchronization. These results highlighted a dynamic interaction between a prefrontal cognitive control network and a hippocampo-cortical memory storage network corroborating previous evidenced by fMRI and EEG studies^13,19,33–35^. It is important to note that the source analysis of Waldhauser et al.^25^ and the present study were applied to different types of signals to localize different types of mechanisms: topographical power spectrum, and phase synchronization measurements of the EEG trials, to investigate oscillatory brain dynamics sources in Waldhauser et al.^25^, and here, the topographical averaged electrical potentials to investigate the sources of the ERP brain responses. Brain dynamics and brain responses can provide non-redundant information characterizing the same process^36–38^. In this way, it seems reasonable to hypothesize that the decrease of theta oscillatory power in the medial temporal lobes and reduced long-range theta phase synchronization characterizing the successful suppression of memory cues might not impeach a concomitant response of the classical motoric cerebral structures as we observed in the present study.

Previous studies have shown significant differences in the amplitudes of the ERPs occurring at earlier latencies than the present periods of interest. Notably, enhanced negativity in the 200–300 ms period for the NT with respect to the T condition and enhanced negativity in the 300–500 ms for subsequently forgotten words only in the case in which participants were specifically asked to block memory retrieval and to focus on the memory cue during the TNT task^31^. Our study could not corroborate these findings, most likely due to different methodological choices. It is important to note that the periods of interest that we selected presented spatially gathered populations of electrodes (and not scattered isolated or preselected ones) in the topographical statistics with the full array of electrodes. This is consistent with the subsequent inverse source modelling that requires the topographical full array of electrical potentials. Thus, the present study cannot exclude that significant differences in the ERPs occur at earlier latencies when focusing the analysis on single isolated electrodes.

Distributed linear solutions have the advantage of not initially preconceiving the number or the location of the calculated generators^27–29^. This method has previously allowed^39^ to estimate the brain sources of the late parietal positivity before 1000 ms (500–800 ms) in a protocol where the TNT and the memory cues were simultaneously presented. The results showed that higher current densities for the T than for the NT were situated in the parietal (BA7), middle temporal (BA21), and occipital (BA19) regions. In addition, the same study showed that such a larger late parietal positivity was associated with the suppression of aversive memories primarily at the right medial and superior frontal gyri. At later latencies (> 1000 ms) and specifically to the word-memory cue the distributed source model revealed that the left primary motor cortex (M1, BA4), thalamus, and premotor cortex (BA6) were the estimated brain sources characterizing the sustained and reduced brain electrical potentials during the inhibition (NT condition) of the target word following its respective memory cue.

Considerable evidence from fMRI studies indicates that the network underlying the inhibition of memory retrieval is right lateralized and includes the frontal cortex (right middle frontal gyrus) which exerts inhibitory modulation over the medial temporal lobe and hippocampus in the NT trials^11,13,33,40^. It is important to keep in mind that fMRI and EEG/ERP are not equivalent but complementary techniques and that their spatial and temporal resolutions are not comparable, thus rendering it difficult to transpose findings. EEG-fMRI fusion approaches have been developed to estimate the brain maps of the EEG and to account for the variable lag structure between electrophysiological and BOLD signals^41^. In this way, Crespo-García et al.^42^ used simultaneous EEG-fMRI recordings and EEG sources modeling to relate previously well-supported EEG/ERP signatures of inhibitory control of unwanted memories to ACC, rDLPFC, and hippocampus brain areas which were highly spatially delimitated by the BOLD signals. It is important to note, that the precise periods of interest used in this multimodal approach were defined from a pooled frontocentral channel (comprising Fz, FC1, and FC2) and that the anatomical regions of interest were predetermined by consistent previous literature. Interestingly, this work showed that the anterior cingulate cortex is in charge of communicating the need for inhibitory control to the rDLPFC through theta oscillations proactively, before 500 ms^43–45^ and late in a reactive way, during 650–1850 ms^25^. In turn, rDLPFC amplifies top-down inhibition over the hippocampus through beta oscillations for achieving successful motivating forgetting.

Source reconstruction models have limitations by definition, as they use mathematical and physiological priors to ensure the uniqueness of the solution; otherwise, more than one brain source configuration could lead to the same topographic distribution^46,47^. Still, source reconstruction from EEG signals is pertinent because, unlike fMRI, EEG is a direct measure of the (global) real electrical brain activity. Moreover, there is electrophysiological evidence from studies simultaneously recording high-density EEG and local field potentials through electrodes implanted in the thalamus and the nucleus accumbens of patients with deep brain stimulation therapy, that EEG contains subcortical activity that can be reconstructed through inverse modeling^48,49^. Hence, when EEG and fMRI strengths and limitations are carefully considered, their combined utilization can go over the time–space resolution correspondence of the results obtained from one technique over the other and strive to reveal parallel mechanisms unified in the same sensory-motor or cognitive process.

As noted before and in contrast to the previous fMRI studies, in the current experiment the T and NT instructions were administered before (1500 ms) the onset of the memory cues. As the participant was already in either the T or the NT mental state well before the memory cue, it seems reasonable to believe that any kind of global or specific motor activation/inhibition which could be related to the TNT instructions per se, was not theoretically attended or solicited by the appearance of the memory cue. Surprisingly, our results showed that the cognitive process related to the inhibition of memory retrieval involved classical motoric cerebral structures. This agrees with previous conceptual propositions that movement is inescapable for understanding cognitive processes^50,51^. There is strong evidence that indicates that the primary motor cortex is an essential player in the final processing stages of a motoric inhibition cortico-subcortical-thalamocortical network involving the prefrontal cortex, premotor cortex (BA6), basal ganglia, thalamus, and finally motor cortex^52–56^. As previously proposed^57^ in the context of movement prevention, increased activity of inhibitory interneurons within M1 may be the mechanism for volitional inhibition of the target word under the NT instruction. In this line, it has been suggested^11^ that the involvement of the basal ganglia is critical for stopping both thoughts and actions and that basal ganglia could be part of a supra-modal network responsible for stopping unwanted processes, including the motor and the cognitive ones. Additionally, the consistent left lateralization in motor control areas observed in the NT condition can be interpreted as an element supporting the neural reuse theory^58^, suggesting that neural circuits established for one purpose (i. e motor response inhibition) can acquire new functions during evolution (i.e. inhibition of linguistic content), without losing its original function. As language is strongly lateralized to the left hemisphere^59–61^, the local left motor inhibition network is activated in order to suppress the verbal processing^62^.

Additionally, in a multimodal fMRI—magnetic resonance spectroscopy study during the TNT paradigm, Schmitz et al.^63^ provided for the first time evidence of GABAergic inhibition of hippocampal activity as a mechanism enabling the prefrontal cortex to suppress unwanted thoughts. In contrast, neither hippocampal GABA nor hippocampal BOLD responses predicted inhibitory control over actions. Moreover, these results showed that while thought suppression was accompanied by hippocampal signal modulations, action stopping was accompanied by left primary motor cortex signal modulations. Therefore, it could be argued that the motoric network that we found might be linked to the phonation motor imagery that would be spontaneously triggered by the presentation of the word memory cue, or to movement suppression of the right-hand, also elicited by the memory-cue as our participants were asked to type their responses during the learning phase (in contrast to most TNT studies requiring words phonation instead). In their original study, Anderson and Green^14^ instructed participants to speak aloud during T trials and remain silent during NT trials. This could imply that the suppression of overt motor response during the NT trials could be responsible for the SIF effect. To rule out this possibility, they introduced a control experiment during which participants had to remain silent for T trials. Thus, they removed phonation in both the T and NT conditions. Similarly, in the current study, participants were asked to remain silent during both T and NT trials.

Interestingly, a recent 7TfMRI study showed that lips pursing during speech production involves the left precentral sulcus^64^; this suggests that motoric engagement may occur during both learning protocols (using phonation or writing). Also, we think that this seems unlikely as a similar phonation motor imagery process or hand movement suppression should concern both the T and the NT conditions, as the word cues are presented in the same way in both conditions and that participants were similarly not required to type anything during the TNT task.

Our source modelling revealed that thinking of the target word was underlain by the involvement of the right prefrontal cortex (BA10), the left cingulate gyrus (BA23) and the left posterior cingulate cortex (BA31). There is a wealth of evidence demonstrating that the prefrontal cortex is involved in memory retrieval^65^. The voluntary recall involves significant regional blood flow increases in the right dorsolateral prefrontal cortex together with the posterior cingulate cortex (BA23) left precuneus (BA7) and right parahippocampal gyrus (BA35/36)^66^. Concretely, right frontal-polar regions are specifically activated in participants who remember a word from a previously learned list by the presentation of a related word stem cue^67^. In this line, Buckner^65,68^ has proposed that the right frontal-polar cortex may be involved in sustained processes, in a type of attentional set or task mode involving multiple individual retrieval events^65,69,70^. This was the case with our present protocol as the TNT phase included a single block of 240 consecutive trials. As previously proposed^65^, the involvement of the right frontal-polar cortex that we observed in the T condition may be interpreted as an indication of the role that it plays in the maintaining of a cognitive set during remembering, by the selection of one mode of processing, in other words the specific cognitive operation or mental strategy, over the other across extended periods of time^65^, as it might be required for the successful execution of the Think and No-Think task. Additionally, the activations observed in the BA23 and BA31 regions specifically during the Think condition are consistent with previous literature, as the posterior cingulate gyrus has been linked to the processing of lexical^71^ and semantic content^72^, as well as the explicit recollection of word stimuli^73^.

Moreover, the TNT phase in our protocol may be seen as a way to approach metacognition which is considered as the capacity to introspectively control one’s own cognitive processes^74,75^, such as during the repetitive switching between thinking or not thinking. Although the identification of the neurophysiological mechanisms underlying metacognition is still in progress, the involvement of the anterior prefrontal cortex in perceptual metacognition is largely recognized^76–78^. More concretely, the right anterolateral prefrontal cortex (BA10) plays a role in metacognitive facets of decision making as during self-report^79^.

Regarding the behavioral analysis, our results did not show significant differences in recall accuracy between the three experimental conditions (NT = B = T). Thus, we did not observe the standard behavioral finding, namely that recall rates drop below baseline for stimuli in the NT condition (NT < B < T). This lack of significance is not unusual, as failures to replicate the suppression-induced forgetting (SIF) effect have already been reported^18,24,80–89^. An explanation for the variability of the SIF effects has been proposed based on the U-shaped curve of the nonmonotonic plasticity hypothesis^81^. Under this vision, the authors indicated that even if the average level of memory activation (across No-Think trials) corresponds to the exact center of the dip in the plasticity curve, any variability around that mean might result in memories falling outside of the dip, thereby reducing the size of the forgetting effect. Our protocol design may be responsible for the lack of significant SIF effect, notably because the trials were consecutively presented in a single block of about thirty minutes of duration without a break, and this could have led to fatigue which is known to weaken SIF^90–92^. Also, it has been shown that increasing post-cue interval duration/response/suppression time from 3 versus 5 s did not increase the forgetting rate of the to-be-forgotten information but on the contrary, it increases its retention^93^. The 5.5 s duration of a whole TNT trial in our protocol could underlie the lack of SIF effect. However, Depue et al.^94^ found significant SIF with similar TNT trial duration. The lack of a significant SIF effect impedes us from elucidating the links between the SIF effect and amplitude variations of ERP and this limitation precludes corroboration of previous evidence provided by Hanslmayr et al.^22^ that the reduction of the late positive component beginning at around 1.6 s after memory cue would reflect item suppression and it would underlie subsequent forgetting.

## Methods

### Ethics declaration

The study was approved by the Ethics Committee of the Brugmann University Hospital, Brussels (Ref. CE2020/204). All participants signed informed consent forms and received a 15€ monetary compensation for their participation. All data was collected, processed, and analyzed following the current General Data Protection Regulation (RGPD) guidelines (https://gdpr-info.eu/)^95^. Study participant names and all other identifiable information has been removed from the paper.

### Participants

Fifty-one healthy subjects (14 males/37 females, 22.8 ± 3.04 (mean±sd) years old) participated in this study. All participants were right-handed and had no neurological or psychiatric history.

Five participants were excluded from data treatment because of the poor quality of the signals or isolated technical issues during the recording sessions.

### Procedure

Several days before coming to the laboratory, participants filled out an online demographic questionnaire via the LimeSurvey platform^96^. On the recording day, participants arrived at the Laboratory of Neurophysiology and Movement Biomechanics (LNMB), received instructions for the experiment, and signed an informed consent form. After being equipped with the EEG cap, they were comfortably seated in front of a computer screen, on which the experimental stimuli were presented via the PsychoPy package^97^. During the memorization-recall phase and the final recall phase, participants used the keyboard and mouse to type and submit their responses. The protocol had a duration of two hours with several breaks during the tasks.

At the end of the experiment, participants were invited to complete the Movement Imagery Questionnaire–Revised Second version (MIQ-RS^98^) before a general final debriefing. The purpose was to investigate the relationship between movement imagery capacity and recall abilities by distinguishing between low and high forgetters, as distinguished by Hanslmayr et al.^22^. As no significant differences were observed in recall accuracy, the correlational analysis was not conducted.

### Experimental protocol

**Figure 4:**
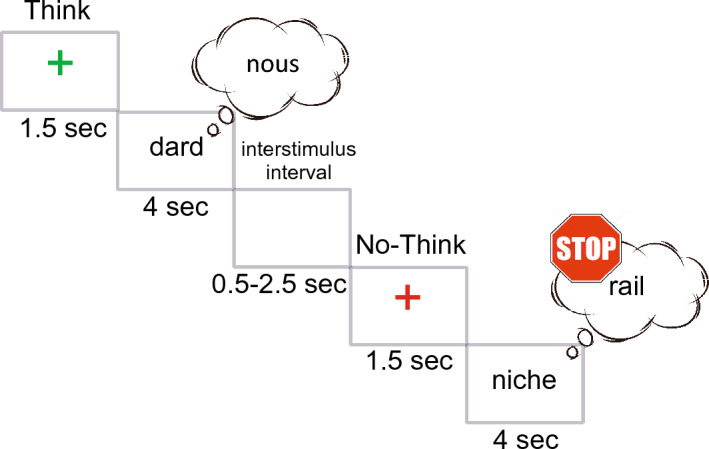
The Think/No-Think experiment, an example of two successive experimental trials, Think and No-Think conditions respectively.

The present Think/No-Think design (Fig. 4) is a modified version of the original paradigm^14^. Conceptually following Hanslmayr et al.^22^, the presentation of the T/NT instructions and memory cues was dissociated by giving the T/NT instructions before presenting the memory cues.

The protocol consisted of three phases: (1) the learning (a memorization-recall) task, (2) the TNT task and (3) the final recall task. Each phase started with 3 practice trials. Concretely, during the learning phase, the complete list of stimuli (45 cue-target word pairs) was displayed one by one in random order at the center of the screen for 4 s. Participants had to observe and memorize them. During the recall task of this learning phase, the first word of each pair (memory cue) was displayed for 2 s and once it disappeared, participants had 8 s to type the second word of the pair (target). Feedback on performance was not provided. If a minimum of 35 correct answers were given, the participant advanced toward the TNT phase, otherwise, the memorization-recall loop ran again. Across all participants, the number of loops required to access the TNT phase was variable, depending on individual memory performance (Mean 3.3 ± 1SD).

The present TNT design consisted of 45-word pairs in the French language. For each participant, 30-word pairs were randomly selected and attributed to the TNT task. The remaining 15-word pairs were attributed to a Baseline condition and were not presented during the TNT task, thus serving as a control condition for the response accuracy dependent variable.

Out of the 30 selected pairs, 15 were randomly attributed to the T condition, while the remaining 15 were attributed to the NT condition. Each stimulus was presented 8 times, resulting in a total of 240 experimental trials (15 pairs T * 8 repetitions = 120 T trials; 15 pairs NT * 8 repetitions = 120 NT trials), presented in a single block, lasting for approximately 30 min. On each trial, the cue word was presented on a grey background at the center of the screen, written in lower-case black Arial font, letter height 0.1. Cue words were displayed continuously on the screen for 4 s, during which participants had to either Think or Not think about the target word. The experimental conditions were set in random order and were announced on a trial-by-trial basis by a fixation cross that was either green (T) or red (NT). The inter-stimulus interval (ISI) was of variable duration between 0.5 and 2 s.

All participants received the same instructions, asking them to apply a direct thought suppression strategy in the NT trials^23,35^: “You have memorized a list of cue-target words. Now, you will see only the cue words displayed at the center of the screen. Your task will be to either think about the target word or not to think about it. You have to inhibit the target, to put it out of your consciousness. When you inhibit the target, please avoid using any replacement strategies, and keep your eyes on the stimulus word”.

During the final-recall phase, all the stimuli, 15 pairs T, 15 pairs NT, and also the 15 pairs from B were used. In this phase, a same-probe memory test was performed by displaying the cue word of each pair at the center of the screen for 2 s. Once it disappeared, participants had 8 s to type the target word that completed the pair.

### Behavioral data analysis

Both for behavioral and EEG data, analysis was conditionalized on initial learning: for each participant, the word pairs that were not correctly recalled during the learning phase (memorization—recall task) were identified and excluded. For the behavioral analysis, to account for inter-subject variability when calculating recall accuracy, the number of correctly recalled items during the final recall phase was expressed in percentages.

Non-parametric Friedman’s test was used to look for significant differences in the recall accuracy between the T, NT, and B conditions. Pairwise comparisons were conducted using an extended version of the Wilcoxon signed-rank test, the Durbin-Conover test, as it allows for multiple pairwise comparisons while controlling the familywise error rate.

### EEG recording parameters

EEG signals were recorded using the ANT Neuro eego™ sport system (ANT Neuro system, *The Netherlands*) at a sampling frequency rate of 2048 Hz. An active-shield cap with 64 Ag/AgCl-sintered ring electrodes (following the 10–20 electrode system placements) and shielded co-axial cables was comfortably adjusted to each participant’s head. The reference electrode was integrated into the EEG cap (Fcz).

### EEG data analysis

Offline data treatment was performed by means of EEGLAB software^99^, ASA software (ANT Neuro system, *The Netherlands*) and in-house MATLAB-based tools. Data were resampled to 512 Hz, low pass (200 Hz) and high-pass (0.1 Hz) filtered, channels with abnormally high amplitude were interpolated, data were re-referenced to REST^100^, visually inspected and portions of data presenting abnormally high amplitude were manually rejected. Artefactual components from eye movement (blinks and horizontal movement) were identified and rejected using the ICALabel plugin of the EEGLAB toolbox.

ERPs were calculated by averaging epochs extracted from − 1000 to 4000 ms of the stimulus event (i.e. memory cue: the appearance of the cue word) in the T and the NT conditions without baseline correction^101–104^. After artifact rejection, we obtained a total of 4158 and 4259 epochs for the T and NT conditions respectively.

The significance in the ERPs (and their topographies) between conditions at the population level was calculated in EEGlab by permutation statistics (*p* < 0.05), corrected for multiple comparisons (ERPs from 64 electrodes) by using the false discovery rate (FDR) method.

### Source analysis

We have estimated brain sources by using the standardized weighted Low Resolution Electromagnetic Tomography (swLORETA) method^26,105^. The method used here is described in detail in Cebolla et al.^36^. From the family of distributed methods, swLORETA allows accurate reconstruction of surface and deep current sources in simulated data even in the presence of noise and when two dipoles are simultaneously active. This is achieved by incorporating a singular value, a decomposition-based lead field weighting that compensates for the varying sensitivity of the sensors to current sources at different depths.

In order to map the generators of the main ERP components evoked by the T and the NT conditions, we computed swLORETA solutions on individuals’ ERP topographies elicited by the onset of the cue word for both conditions, within the following time intervals of interest: 1000–1150 ms, 1200–1350 ms, 1550–1700 ms, 1850–2000 ms.

As part of the LORETA inverse solution analysis in ASA software, the data were automatically re-referenced to the average reference and the Boundary Element Model (BEM) was formerly used for solving the forward problem^106^. The inverse solution was restricted to the grey matter based on the probabilistic brain tissue maps available from the MNI^107^. Voxels (10.00-mm grid size) and the electrode arrangement were placed in registration with the Collins 27 MRI produced by the Montreal Neurological Institute^107^.

For the statistical maps, the current density of every voxel of every participant and condition (T and NT) was divided by the mean current density value of all voxels of the same participant and condition. This gave us a normalized inverse solution in which a voxel value greater than 1 indicates greater activity than the mean. In the next step, 2000 permutations were carried out to obtain a normalized inverse solution population map for every condition. After the homogeneity of variance and normal distribution were confirmed by a Levene test and by a Kolmogorov–Smirnov test respectively, paired t-tests (*p* < 0.05) were calculated between the T and NT conditions. If homogeneity and normality criteria were not met, a Wilcoxon signed-rank test was used for the comparison. Both T > NT, and NT > T contrasts were computed. All time periods and contrasts, except 1850–2000 ms, had a normal distribution.

The final Talairach coordinates^108^ (directly accessible for every voxel in ASA software) reported in the results section correspond to the clusters’ maxima values of the final statistical maps. In addition, the corresponding Brodmann areas inside the cortical mantle are provided (talairach.org from Research Imaging Institut).

## Conclusion

The aim of this study was to investigate the neural correlates and the underlying brain generators elicited by the word memory cue in the Think/No-Think paradigm in a high-density EEG array in 46 participants and with a distributed method that does not preconceive any particular location within the gray matter volume. We corroborate previous findings showing sustained and reduced brain electrical frontal and parietal late potentials. Such reduction was significant 1000 ms after the memory cue onset and was long-lasting. Our findings showed that cognitive processes related to the inhibition of memory retrieval involved classical motoric cerebral structures with the left primary motor cortex (M1, BA4), thalamus, and premotor cortex (BA6). Also, the right frontal-polar cortex was involved in the Think condition, which we interpreted as an indication of its role in maintaining a cognitive set during remembering, by the selection of one mode of processing, in other words the specific cognitive operation or mental strategy, over the other one across extended periods of time as it might be necessary for the successful execution of the Think/No-Think task.

### Supplementary Information

Supplementary Information 1.

Supplementary Information 2.

## Data Availability

The datasets generated during and/or analyzed during the current study are available from the corresponding author upon reasonable request.
