# Supplementary material for: Long-lasting negativity in the left motoric brain structures during word memory inhibition in the Think/No-Think paradigm
Source: Sci Rep. 2024 May 13;14:10907. doi: 10.1038/s41598-024-60378-y (PMC11091218; doi:10.1038/s41598-024-60378-y)
Supplement: Supplementary file 1 — Supplementary Information 1. [file 41598_2024_60378_MOESM1_ESM.pdf]

Supplementary Information

Long-lasting negativity in the left motoric brain structures during word memory inhibition in the Think/No-Think paradigm

Viktoriya Vitkova[1,2], Dominique Ristori[1], Guy Cheron[1], Ariane Bazan[1,2] and Ana Maria Cebolla*[1]

1. Laboratory of Neurophysiology and Movement Biomechanics, Université Libre de Bruxelles, Brussels, Belgium
2. InterPsy Laboratory, Université de Lorraine, Nancy, France

❖ Corresponding author : Ana.Maria.Cebolla.Alvarez@ulb.be

Supplementary Table S1. Descriptive statistics of recall accuracy during the memorization phase and the final recall phase.

	Recall accuracy memorization phase	Recall accuracy memorization phase (%)	Recall accuracy final recall (%)
N	46	46	46
Mean	38.30	85.20	95.80
Median	38.00	84.40	97.10
Standard deviation	2.69	5.99	4.17
Minimum	35.00	77.80	86.10
Maximum	44.00	97.80	100.00

Supplementary Table S2. Frequencies of the recall accuracy scores during the memorization phase.

Recall accuracy observed score	Counts	% of Total	Cumulative %
35	8	17.40	17.40
36	7	15.20	32.60
37	4	8.70	41.30
38	6	13.00	54.30
39	7	15.20	69.60
40	4	8.70	78.30
41	5	10.90	89.10
42	1	2.20	91.30
44	4	8.70	100.00

Supplementary Table S3. Descriptive statistics on the recall accuracy during final recall expressed in percentages to account for intersubject variability in performance during memorization phase.

	% No-Think	% Baseline	% Think
N	46	46	46
Mean	95.60	95.40	96.80
Median	100.00	100.00	100.00
Standard deviation	6.95	7.12	5.60
Minimum	72.70	75.00	81.80
Maximum	100.00	100.00	100.00

Supplementary Information

Supplementary Table S4. Non-parametric repeated measures ANOVA (Friedman test) on the conditionalized recall accuracy data at final recall expressed in percentages. A) Friedman test. B) Pairwise comparisons (Durbin-Conover).

Supplementary Table S4 A. Friedman test

χ^2	df	p
0.62	2	0.73

Supplementary Table S4 B. Pairwise comparisons (Durbin-Conover)

	Statistic	p
No-Think vs Baseline	0.28	0.78
Think vs Baseline	0.49	0.62
Think vs No-Think	0.77	0.44

Supplementary Table S5. Non-parametric repeated measures ANOVA (Friedman test) on the non-conditionalized recall accuracy data at final recall expressed in number of correctly recalled words. A) Friedman test. B) Pairwise comparisons. C) Descriptives.

Supplementary Table S5 A. Friedman test

χ^2	df	p
0.33	2	0.84

Supplementary Table S5 B. Pairwise comparisons (Durbin-Conover)

No-Think vs Baseline	0.28	0.77
Think vs Baseline	0.57	0.56
Think vs No-Think	0.28	0.77

Supplementary Table S5 C. Descriptives

	Mean	Median
No-Think	12.30	13.00
Baseline	12.10	12.00
Think	12.30	12.00
